# Expanded Clinical Spectrum of Autosomal-Dominant STT3A-CDG

**DOI:** 10.3390/biom16030418

**Published:** 2026-03-12

**Authors:** Hamdan Al-Shahrani, Evelin Szabó, Caroline Staccone, Georgia MacDonald, Yutaka Furuta, Daniel Schecter, Andrew C. Edmondson, Anne McRae, Josh Baker, Eva Morava, Rory J. Tinker

**Affiliations:** 1 Department of Genetics and Genomic Sciences, Icahn School of Medicine at Mount Sinai, New York, NY 10029, USA; 2 Department of Human Anatomy, University of Pécs Medical School, 7624 Pécs, Hungary; 3 Department of Human Genetics, School of Medicine, Emory University, Atlanta, GA 30332, USA; 4 Department of Pediatrics, Division of Human Genetics, Children’s Hospital of Philadelphia, Philadelphia, PA 19104, USA; 5 Division of Genetics, Ann & Robert H. Lurie Children’s Hospital of Chicago, Chicago, IL 60611, USA; 6 Department of Biophysics, University of Pécs Medical School, 7624 Pécs, Hungary

**Keywords:** STT3A, congenital disorder of glycosylation, dominant-negative, oligosaccharyltransferase, genotype–phenotype correlation

## Abstract

*STT3A* encodes the catalytic subunit of the oligosaccharyltransferase A (OST-A) complex and is classically linked to severe autosomal-recessive congenital disorder of glycosylation (CDG). To define the distinct autosomal-dominant disorder, we reviewed all published cases and integrated three previously unpublished individuals from the CDG natural history study. Across 21 individuals, abnormal transferrin glycosylation was present in nearly all individuals (20/21), and subtle facial dysmorphism was common (18/21). Neurodevelopmental involvement was frequent, including motor delay (13/21), learning difficulties (13/21), speech delay (12/21), and intellectual disability (10/21). Musculoskeletal manifestations were also common, including skeletal abnormalities (12/21), short stature (11/21), muscle cramps (8/21), and early-onset osteoarthritis in adults (6/21). Less frequent features included congenital heart defects (5/21) and coagulation factor deficiency (5/21). Importantly, the newly reported individuals expand dominant STT3A-CDG with previously unreported features, including anorectal malformation, morbid obesity, and clinically significant bleeding diathesis with von Willebrand factor and factor VIII deficiency. Biochemical signatures ranged from classic type I transferrin patterns to subtle or atypical abnormalities, emphasizing that near-normal transferrin testing does not exclude the diagnosis. Variants clustered in conserved catalytic regions, with recurrent p.Arg405 across de novo, inherited, and mosaic cases supporting a mutational hotspot and likely dominant-negative mechanism.

## 1. Introduction

Congenital disorders of glycosylation (CDGs) are a heterogeneous group of inherited metabolic diseases caused by defects in the synthesis, processing, or attachment of glycans to proteins and lipids, leading to multisystem disease most often characterized by neurodevelopmental impairment, growth abnormalities, and variable hepatic, endocrine, hematologic, and immune involvement; however, their clinical heterogeneity and limited disease-specific biomarkers frequently result in substantial diagnostic delay [1,2,3,4,5,6]. In this context, *STT3A* gene (MIM 601134), located on 11q24.2, encodes the catalytic subunit of the OST-A complex responsible for co-translational N-glycan transfer in the endoplasmic reticulum [7,8,9]. *STT3A* was long believed to cause disease only through autosomal recessive inheritance (MIM 615596), based on early reports of consanguineous families with biallelic missense variants that reduced *STT3A* abundance, impaired N-glycosylation, and produced a severe neurodevelopmental phenotype [10,11]. Recent genomic and functional studies, however, have revealed a second mechanism: heterozygous, catalytic-site missense variants that exert a dominant-negative effect on OST-A, establishing autosomal dominant STT3A-CDG (MIM 619714) [12]. This shift demonstrates that *STT3A* defects span both recessive and dominant inheritance and raises new questions regarding penetrance, pathogenic mechanisms, and clinical variability [13].

Dominant STT3A-CDG is characterized by missense variants that alter residues essential for substrate binding or catalytic function, impairing oligosaccharide transfer despite near-normal protein levels. Functional studies (including *S. cerevisiae* complementation, OST substrate assays, and patient fibroblast analyses) confirm reduced N-glycosylation and a characteristic type I transferrin pattern [12]. Clinically, individuals present with developmental delay, craniofacial differences, short stature, musculoskeletal anomalies, and EEG abnormalities, though severity varies widely [12,14,15]. The coexistence of both dominant and recessive pathogenic mechanisms within *STT3A* underscores the sensitivity of OST-A to even partial catalytic disruption. Yet the literature remains limited, and the full phenotypic spectrum, natural history, and biochemical consequences of heterozygous *STT3A* variants are not well defined. More broadly, even as diagnostic yield improves with next-generation sequencing, large rare-disease cohorts continue to show that a substantial fraction of inherited metabolic and rare disorders are missed by traditional screening approaches and are increasingly identified through integrated genomic and multidisciplinary evaluation [16,17,18].

The aim of this study is to synthesize current knowledge of dominant STT3A-CDG, integrate newly described cases and patients from the CDG natural history study with existing mechanistic and clinical data, and clarify how heterozygous catalytic-site variants disrupt OST-A function [19]. We hypothesize that dominant STT3A-CDG is an under-recognized glycosylation disorder with broader phenotypic variability than currently appreciated. Our objectives are to (1) summarize the molecular spectrum underlying the dominant-negative disruption of *STT3A*, (2) delineate the emerging clinical phenotype and, and (3) identify key gaps that should guide future diagnostic, functional, and natural history studies.

## 2. Materials and Methods

### 2.1. Data Sources

We conducted a targeted literature review to identify all published STT3A-CDG cases using terms including “*STT3A*,” “congenital disorder of glycosylation,” “CDG type Iw,” “dominant-negative glycosylation,” and “oligosaccharyltransferase.” PubMed, Embase, and OMIM were searched without date or language limits, and reference lists were screened to ensure complete ascertainment.

### 2.2. Natural History Study

We also identified three previously unpublished individuals with heterozygous *STT3A* variants from the Frontiers of Congenital Disorders of Glycosylation Consortium (FCDGC) natural history study (IRB: 19-016991; ClinicalTrials.gov NCT04199000), drawn from 410 participants in the NIH RDCRN RedCAP database [19]. All three were enrolled at Mount Sinai (New York, NY, USA) within the past year. Data were collected prospectively during study visits and supplemented by retrospective chart review under the IRB-approved protocol.

### 2.3. Phenotypic Extraction and Transferrin Glycoform Reference Intervals

For published and FCDGC cases, phenotypes were abstracted using a structured framework and mapped to standardized domains (neurodevelopment, growth, craniofacial, musculoskeletal, EEG/neuroimaging, ophthalmologic/systemic findings, and biochemical glycosylation markers). Molecular details (inheritance, variant position, catalytic-site proximity, and predicted functional impact) were recorded where available. Terminology was harmonized across reports to enable comparisons and qualitative synthesis of recurrent features, variability, and genotype–phenotype patterns [20,21]. Transferrin glycoform analysis and ApoC-III isoform profiling were abstracted from clinical laboratory reports and prior publications; testing was performed in CLIA-certified laboratories using standard methods (e.g., isoelectric focusing/capillary electrophoresis with immunofixation and/or mass spectrometry). Because assay platforms and cutoffs varied across sources, we report results as issued by the performing laboratories. Where available, transferrin results are summarized using laboratory-reported glycoform ratios (e.g., mono-oligo/di-oligo, a-oligo/di-oligo, tri-sialo/di-oligo), which reflect enrichment of under-glycosylated transferrin species relative to the normally glycosylated di-oligo fraction; elevations above each laboratory’s upper reference limit were interpreted as consistent with a type I CDG pattern.

### 2.4. Variant Curation and Gene-Structure Visualization

All reported autosomal-dominant *STT3A* variants (literature + this study) were curated (cDNA/protein change, inheritance, clinical/biochemical annotations) and standardized to NM_001278503.1 (hg38) using HGVS nomenclature and HPO terms [22,23]. Recurrent variants were retained with provenance but collapsed to a single genomic position for visualization. *STT3A* exon–intron structure was derived from UCSC hg38 RefSeq (TxDb.Hsapiens.UCSC.hg38.refGene), transcript coordinates were reconstructed from exon intervals, and variants were projected onto the gene model. Schematics were generated in R (v4.5.2) using ggplot2 (exons as boxes, introns as segments, variants as lollipops) and exported at publication resolution.

## 3. Results

### 3.1. Initially Reported Autosomal-Dominant STT3A-CDG

The initial report described 16 individuals from 9 families with heterozygous *STT3A* missense variants, establishing an autosomal-dominant CDG with recurrent features including short stature, macrocephaly, craniofacial dysmorphism, skeletal anomalies, hypertonia, and muscle cramps, with intellectual disability in 50% [12]. Pathogenic variants clustered at the catalytic site, and functional studies supported a dominant-negative mechanism (abnormal glycosylation despite normal *STT3A* expression; supportive yeast data). Abnormal transferrin glycoforms were notable and helped distinguish dominant STT3A-CDG from recessive STT3A-CDG.

### 3.2. Recently Reported Individuals Expanding the Phenotypic and Mechanistic Spectrum

Subsequent reports have described two additional individuals with de novo heterozygous *STT3A* variants, further expanding the recognized clinical and mechanistic spectrum of autosomal-dominant STT3A-CDG (Table 1, main text). These individuals shared core features with the originally reported cohort, including developmental delay, short stature, craniofacial dysmorphism, and epilepsy or abnormal EEG findings, while also exhibiting broader neurobehavioral phenotypes such as autism spectrum disorder and attention-deficit/hyperactivity disorder in some cases. Molecular and functional data from these studies further strengthened the dominant disease model, demonstrating variant-specific disruption of glycosylation sites, reduced *STT3A* protein levels in vitro, and in vivo validation using heterozygous zebrafish knockdown models that recapitulated key patient phenotypes, including craniofacial and skeletal anomalies, developmental delay, behavioral alterations, and electrophysiological abnormalities.

### 3.3. Unpublished Cases Expanding the Phenotypic Spectrum of Dominant STT3A-CDG from the Natural History Study

Patient 1. An 18-month-old female with a de novo heterozygous pathogenic *STT3A* variant (c.1213C>T; p.Arg405Cys) was diagnosed at birth and followed for growth and developmental concerns. She had an anorectal malformation and a sacral dimple. Family history was noncontributory. Her course included short stature, feeding intolerance, chronic otitis media and developmental delay (early speech delay requiring therapy). Cardiac findings included a resolved PFO and a bicuspid aortic valve. By 18 months she had achieved major motor milestones without regression. Growth remained <10th percentile. Laboratory testing showed mild anemia, transient hypoglycemia, and low factor VIII activity. CDG biomarker testing demonstrated elevated mono-oligo/di-oligo and asialo/di-oligo ratios, consistent with a type-I CDG pattern. Examination showed good social engagement, preserved tone, and a stable gait with mild truncal sway, along with dysmorphic features (hypertelorism, flat nasal bridge, prominent glabella, smooth philtrum). Overall, her presentation is consistent with the expanding phenotype of dominant STT3A-CDG; the anorectal malformation may represent a rare or previously underrecognized feature. Her Nijmegen Progression CDG Rating Scale score was 9, indicating mild severity.

Patient 2. A 10-year-old male twin B born premature at 30 weeks of gestation who had a multisystem disease including spontaneously resolved ASD/VSD, repaired laryngeal cleft, early-life seizures (with normal brain MRI), recurrent respiratory/skin infections, easy bruising, livedo reticularis, scoliosis, and severe recurrent muscle cramps. The spine MRI showed a small thoracic syrinx. He had early growth delay followed by rapid pubertal weight gain (weight 99th percentile; height 54th), persistent learning difficulties and behavioral abnormalities requiring medication. Family history was noncontributory. Chromosomal microarray was normal. Whole-exome sequencing identified a heterozygous *STT3A* likely pathogenic variant (c.323T>A; p.Ile108Asn). Biochemical testing showed mild ApoC-III hypo-glycosylation with normal transferrin glycosylation (mono/di-oligo 0.03; asialo/di-oligo 0.000; trisialo/di-oligo 0.02), consistent with a atypical biochemical profile despite a clear molecular diagnosis of autosomal-dominant STT3A-related CDG. The Nijmegen Progression CDG Rating Scale score was 20 (moderate severity).

Patient 3. An 8-year-old female was diagnosed with autosomal-dominant STT3A-CDG during evaluation for lifelong growth delay. Born at 27 weeks, she had early feeding intolerance (resolved) but ongoing cyclic vomiting and global developmental delay. Additional features include short stature on growth hormone, hypotonia with mild coordination difficulties, speech/cognitive delays, ADHD, and anxiety. Dysmorphology included hypertelorism with vertically narrow palpebral fissures, bushy eyebrows, and a bulbous nasal tip with a midline nasal dimple, as well as a prominent forehead and mild facial asymmetry. Ophthalmology showed mild hyperopia; cardiac workup was unremarkable aside from a resolved PFO. She has a significant bleeding phenotype with very low vWF activity (<20%) and factor VIII (10%) with prolonged aPTT, consistent with reported STT3A-CDG-associated coagulopathy. She has had no regression and no confirmed seizures. Biochemical testing supported a CDG type I transferrin pattern. Serum transferrin glycoform analysis was interpreted as abnormal and suggestive of a CDG type I profile, with elevated mono-oligo/di-oligo ratio (0.24) and a-oligo/di-oligo ratio (0.017), while tri-sialo/di-oligo ratio was 0.02. Apolipoprotein CIII ratios were also reported (Apo CIII-1/CIII-2 2.55; Apo CIII-0/Apo CIII-2 0.48). Exam showed short stature with preserved mobility. She was diagnosed with a heterozygous likely pathogenic *STT3A* variant (c.1213C>T; p.Arg405Cys), inherited from her mother with evidence of maternal mosaicism on Genome sequencing. The Nijmegen Progression CDG Rating Scale: 14 (mild severity).

### 3.4. Genotype Summary of Autosomal-Dominant STT3A-CDG

**Figure 1:**
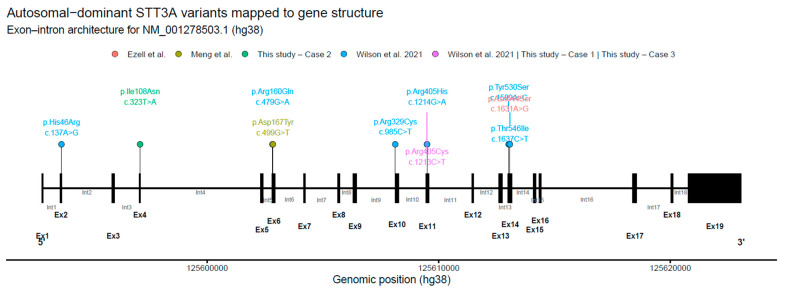
Autosomal-dominant *STT3A* variants mapped to gene structure. Schematic representation of the exon–intron architecture of *STT3A* based on transcript NM_001278503.1 (hg38). Exons are shown as filled boxes and introns as connecting lines, with exon and intron numbers indicated. Missense variants associated with autosomal-dominant disease are displayed as lollipop markers above the transcript, with labels indicating the corresponding protein and cDNA changes. Recurrent variants are plotted once at their genomic position, with provenance summarized in accompanying tables. Variant distribution highlights clustering within exons encoding the catalytic region of *STT3A* [12,14,15].

Across published reports and the present study, we identified multiple heterozygous missense variants in *STT3A* reported in individuals with an autosomal-dominant congenital disorder of glycosylation phenotype (Table 1; Supplementary Table S2; Figure 1). The variants affect highly conserved residues and, based on prior structural and functional work, are consistent with involvement of sequence elements important for *STT3A* catalytic function within the oligosaccharyltransferase complex. Most variants were reported as de novo, although several were inherited, including variants associated with milder or atypical biochemical phenotypes (Supplementary Table S2). A recurrent p.Arg405 variant was observed in multiple unrelated individuals, including de novo, inherited, and parental mosaic cases, suggesting a mutational hotspot and variable inheritance mechanisms (Supplementary Table S2; Figure 1). While variants are distributed across multiple exons, they appear to cluster in regions likely to be functionally important, compatible with a shared pathogenic mechanism. When mapped onto the exon–intron structure of *STT3A*, disease-associated variants were not evenly distributed and were enriched in coding exons corresponding to the central portion of the transcript (Figure 1). Collectively, these observations support *STT3A* as a gene in which specific missense variants can be associated with dominant disease and variable expressivity.

### 3.5. Phenotypic Summary of Dominant STT3A-CDG from All Published and Unpublished Cases

Across a combined cohort of 21 unrelated families, including all published cases and the new cases reported here, with autosomal dominant *STT3A* associated congenital disorder of glycosylation, a variable but recognizable multisystem phenotype was observed (Table 2). Biochemically, abnormal serum transferrin glycosylation consistent with a type I CDG was identified in nearly all individuals (20 of 21), and fibroblast studies uniformly demonstrated glycoprotein hypoglycosylation when tested (Table 2). Phenotypically, first, subtle facial dysmorphism was present in most individuals (18 of 21), with recurrent features including high anterior hairline, short palpebral fissures, and thin upper lip vermilion. Second, neurodevelopmental involvement was common, with motor delay (13 of 21), learning difficulties (13 of 21), and speech delay (12 of 21) frequently reported; intellectual disability was present in approximately half of the cohort (10 of 21). Third, musculoskeletal manifestations were prominent, including skeletal abnormalities (12 of 21), muscle cramps (8 of 21), and early-onset osteoarthritis in several adults (6 of 21). Short stature (11 of 21) and macrocephaly (7 of 21) were also observed. Seizures and behavioral diagnoses were infrequent. Stratification by inheritance demonstrated substantial phenotypic overlap between de novo and inherited autosomal-dominant cases (Supplementary Table S3). Neurodevelopmental features were more frequently reported among individuals with de novo variants, whereas musculoskeletal and biochemical findings were shared across both groups. Given the limited cohort size and potential ascertainment bias, these differences should be interpreted cautiously.

Compared with autosomal-recessive STT3A-CDG, the autosomal-dominant form is distinguished by heterozygous missense variants clustered in highly conserved catalytic/active-site residues, consistent with a dominant-negative, qualitative disruption of OST-A enzymatic activity despite near-normal *STT3A* mRNA/protein levels (Table 3). In contrast, recessive STT3A-CDG reflects loss-of-function with reduced *STT3A* abundance/function and a quantitative reduction in overall OST-A activity, and typically presents as a more severe, early-onset, relatively homogeneous neurodevelopmental disorder with broader multisystem involvement (including prominent feeding difficulties and reported factor VIII/von Willebrand factor deficiency). Overall, dominant STT3A-CDG shows marked inter-individual variability with prominent musculoskeletal features and variable neurodevelopmental impact, whereas the recessive phenotype is generally more consistently severe neurologically.

## 4. Discussion

In this study, we integrate all published evidence on autosomal-dominant STT3A-related congenital disorder of glycosylation with three newly described individuals from the CDG natural history study, expanding both the genotypic and phenotypic spectrum of dominant STT3A-CDG. Our novel cases support that heterozygous missense variant in the catalytic core act through a dominant-negative mechanism, disrupting OST-A-mediated N-glycosylation [24]. Importantly, we identify novel phenotypic features in autosomal-dominant STT3A-CDG that broaden the recognized organ system involvement beyond prior reports. In addition to cardiac malformations and morbid obesity, which were not emphasized in earlier descriptions, we further extend the phenotype to include anorectal malformation, clinically significant bleeding diathesis characterized by marked reductions in von Willebrand factor activity and factor VIII levels, and behavioral abnormalities including attention-deficit/hyperactivity disorder and anxiety. These findings expand the clinical boundaries of dominant STT3A-CDG to encompass congenital structural anomalies, more severe hematologic involvement than previously appreciated, and neurobehavioral manifestations, thereby refining both diagnostic recognition and anticipatory management of affected individuals (Table 2) [9]. We also observed some variability in biochemical signatures: predominantly classic Type I patterns, with infrequent subtle or atypical deviations [25,26]. Recurrent involvement of p.Arg405, including de novo occurrence, inheritance, and parental mosaicism, highlights a mutational hotspot and underscores variable expressivity and penetrance [27,28].

In the context of prior reports, the original description of dominant STT3A-CDG emphasized short stature, craniofacial differences, macrocephaly, skeletal anomalies, and variable neurodevelopmental involvement, with variants localizing to the catalytic site [12,14,15]. Subsequent publications added neurobehavioral phenotypes and provided in vivo validation in zebrafish models [15]. Our expanded cohort is broadly concordant with these observations, but suggests a wider clinical range, including congenital malformations and clinically meaningful coagulation abnormalities. We also confirm that transferrin testing can be insensitive in most dominant cases; but in addition to very mild, a normal transferrin profile does not exclude the diagnosis when clinical and genetic findings are supportive.

Compared with autosomal-recessive STT3A-CDG, the dominant disorder differs substantially, as recessive STT3A-CDG typically reflects biallelic variants that reduce *STT3A* abundance, produce more global impairment of N-glycosylation, and cause a more severe, early-onset neurodevelopmental phenotype. While both conditions reflect OST-A dysfunction, the clinical consequences diverge in severity and consistency: recessive disease is generally more severe and homogeneous, whereas dominant disease shows greater inter-individual variability and survival into adulthood with relatively preserved function in some individuals [10]. Together, these patterns support a model in which quantitative loss of *STT3A* activity drives severe multisystem disease, while qualitative catalytic disruption in the presence of wild-type protein produces a dominant-negative, context-dependent glycosylation defect.

Several limitations should be acknowledged. Case numbers remain modest, limiting genotype–phenotype inference. Phenotypes were derived partially from heterogeneous sources (literature review), increasing reporting and ascertainment bias; however, the newly described individuals were ascertained through and systematically phenotyped as part of a CDG natural history study, providing more standardized clinical characterization. Biochemical evaluation was not standardized across individuals, and mild abnormalities may be missed by transferrin-only assays. Adult natural history data remain sparse, limiting conclusions about long-term morbidity and late-onset complications.

Despite these constraints, the work has diagnostic and research implications. Dominant STT3A-CDG should be considered in individuals with unexplained short stature, skeletal anomalies, neurodevelopmental differences, bleeding diathesis, or multisystem involvement. Even when transferrin studies are normal. Mechanistically, *STT3A* defect provides a clear example of dominant-negative enzymatic dysfunction within a multiprotein complex, with broader relevance to glycosylation biology. Future priorities include standardized biochemical phenotyping, functional validation of additional variants, and prospective natural history studies to clarify disease trajectory, penetrance, and late complications. The potential role of modifier genes and environmental factors in shaping expressivity also warrants investigation.

## 5. Conclusions

Autosomal-dominant STT3A-CDG is an under-recognized, variably expressive multisystem glycosylation disorder driven by dominant-negative catalytic-site missense variants, and our integrated cohort broadens its diagnostic spectrum, highlighting that normal or subtle transferrin profiles do not exclude the diagnosis and that congenital malformations and clinically significant bleeding can be key clues.

## Figures and Tables

**Table 1:** Clinical and molecular features of individuals with autosomal-dominant *STT3A* variants from new publications and clinical reports. Summary of clinical, biochemical, and molecular findings in individuals with heterozygous *STT3A* missense variants associated with autosomal-dominant congenital disorder of glycosylation. Cases include previously published individuals and those from the present study, with variant nomenclature based on transcript NM_001278503.1. Molecular testing methods and inheritance patterns are indicated. Transferrin (Tf) glycoform analysis is shown where available. Clinical features are reported as present (+), absent (−), or unknown (?). Abbreviations: AD, autosomal dominant; ASD, atrial septal defect; AV, aortic valve; ES, exome sequencing; GS, genome sequencing; IUGR, intrauterine growth restriction; PFO, patent foramen ovale; Tf, transferrin; VSD, ventricular septal defect.

	Ezell et al. [14]	Meng et al. [15]	Unreported| (This Study)	Unreported (This Study)	Unreported (This Study)
Sex	M	F	M	F	F
Consanguinity	−	−	−	?	−
Ancestry	European	Chinese	European	European	European
Variant cDNA	c.1631A>G	c.499G>T	c.323T>A	c.1213C>T	c.1213C>T
Variant protein	p.Asn544Ser	p.Asp167Tyr	p.Ile108Asn	p.Arg405Cys	p.Arg405Cys
Mode of inheritance	de novo (AD)	de novo (AD)	Paternal (AD)	de novo (AD)	Maternal mosaic (AD)
Molecular test	GS	ES reanalysis	ES	ES	GS
Age at CDG diagnosis	16 years	5 years	10 years	18 months	8 years
Biochemical studies	↑ Mono-oligo/Di-oligo Tf (0.15); normal A-oligo/Di-oligo	−	−	↑ Mono-oligo/Di-oligo (0.40); ↑ A-oligo/Di-oligo (0.018)	Normal transferrin profile
Gestational weeks	Full-term	Full-term	30 weeks	Full-term	27 weeks
IUGR	+	−	−	−	−
Birth weight (g)	2693	2150	−	−	1040
Birth length (cm)	48	48	−	−	32
Head circumference	Macrocephaly	Normal	−	−	Normal
Short stature	+	+	−	+	+
Failure to thrive	+	−	−	−	−
Dysmorphic features					Hypertelorism; narrow vertical palpebral fissures; bushy brows; bulbous nasal tip + midline nasal dimple.
Long face	−	+	−	−	−
High anterior hairline	+	−	−	−	−
Short palpebral fissures	+	−	−	−	−
Hypertelorism	+	−	−	+	−
Eye shape abnormalities	Down-slanting, slit-like	Almond-shaped	−	−	Mild esotropia
Wide nasal bridge	+	−	−	+	−
Long/protruding ears	−	+	−	−	−
Thin upper lip vermilion	+	+	−	?	−
Smooth/flat/long philtrum	+ (Flat)	+ (Long)	−	+ (Smooth)	−
Prognathism	+ (Maxillary)	?	−	−	−
Inverted nipples	+	−	−	−	−
Abnormal fat distribution	−	−	−	−	−
Other facial features	Long mouth, attached earlobes	Midfacial hypoplasia, prominent forehead	−	−	Prominent forehead
Neurological					
Motor developmental delay	+	+	+	+	+
Speech delay	+	+	+	+	+
Learning problems	+	+	+	?	+
Seizures	+	−	+	−	−
Abnormal muscle tone	Hypotonia	−	−	−	Hypotonia
Behavioral abnormalities	Autism, ADHD, anxiety	−	Aggression	−	ADHD, anxiety
Brain MRI	Normal	Arachnoid cyst, ventriculomegaly	Normal	−	Not performed
Spinal	−	−	Thoracic syrinx	Sacral dimple	−
Musculoskeletal					
Skeletal anomalies	Maxillary prognathism, syndactyly, pes planus	−	−	−	Motor coordination disorder
Osteoarthritis	−	−	−	−	−
Scoliosis	−	+ (mild)	+	−	−
Long bone abnormalities	−	−	−	−	−
Muscle cramps	−	−	+	−	−
Muscle hypertrophy	−	−	−	−	−
Intellectual disability	+	−	−	−	Mild
Eye	−	Refractive errors	−	−	Hyperopia
ENT	High-arched palate	−	Laryngeal cleft	−	−
Cardiovascular	Prominent ascending aorta/root	−	Small ASD, VSD	PFO, bicuspid AV	Resolved PFO
Hematologic	−	−	Easy bruising	Mild anemia	Low vWF, low Factor VIII
Recurrent infections	−	−	+	+	−
Dermatologic	−	−	Livedo reticularis	−	−
Genitourinary	−	−	−	Anorectal malformation	−
Laboratory abnormalities					
Increased PT/APTT	−	−	−	−	+ (APTT)
Decreased coagulation factors	−	−	−	Low FVIII	Low FVIII, low vWF
Increased transaminases	−	−	−	−	−
Abnormal hormone levels	−	−	−	−	On growth hormone for short stature

**Table 2:** Phenotypic features of autosomal-dominant STT3A-CDG. Summary of clinical, biochemical, and molecular features observed in a combined cohort of 21 unrelated individuals with autosomal-dominant STT3A-associated congenital disorder of glycosylation. Features are reported as the number of affected individuals out of the total cohort (n of 21). Absence of a feature does not imply systematic assessment in all individuals.

Feature	Observed Frequency (n of 21)	Comment	Novel in This Study
Growth/Prenatal			
Intrauterine growth restriction	3 of 21		No
Short stature	11 of 21	Childhood and adulthood	No
Failure to thrive	3 of 21	Primarily pediatric	No
Macrocephaly	7 of 21	Often progressive	No
Morbid obesity	1 of 21	Rapid pubertal weight gain	Yes
Dysmorphic features			
Any facial dysmorphism	18 of 21	Typically subtle	No
High anterior hairline	10 of 21	Recurrent feature	No
Short palpebral fissures	7 of 21	Variable	No
Wide nasal bridge	7 of 21	Mild–moderate	No
Thin upper lip and/or philtrum abnormality	9 of 21	Common	No
Prognathism	6 of 21	Maxillary predominance	No
Protruding or low-set ears	6 of 21	Variable	No
Inverted nipples	6 of 21	Distinctive but not universal	No
Abnormal fat distribution	4 of 21	Hip or truncal fat pads	No
Neurodevelopmental			
Motor developmental delay	13 of 21	Usually mild–moderate	No
Speech delay	12 of 21	Some non-verbal	No
Learning difficulties	13 of 21	Broad spectrum	No
Intellectual disability	10 of 21	Mild to severe	No
Seizures	4 of 21	Not a core feature	No
Abnormal muscle tone	7 of 21	Hyper- or hypotonia	No
Behavioral/Psychiatric			
ADHD and/or anxiety	5 of 21	Mostly pediatric	No
Aggressive behavior	3 of 21	Rare	No
Musculoskeletal			
Skeletal abnormalities	12 of 21	Metaphyseal/vertebral	No
Scoliosis	5 of 21	Usually mild	No
Osteoarthritis (adult)	6 of 21	Early onset	No
Muscle cramps	8 of 21	Adolescence/adulthood	No
Muscle hypertrophy	4 of 21	Often with cramps	No
Cardiovascular			
Congenital heart defect	5 of 21	ASD, VSD, PFO, bicuspid AV	No
Genitourinary/Congenital anomalies			
Anorectal malformation	1 of 21	Not previously reported	Yes
Hematologic			
Easy bruising/bleeding	4 of 21	Variable	No
Coagulation factor deficiency	5 of 21	FVIII and/or vWF	Previously rare
Clinically significant bleeding diathesis	3 of 21	Marked vWF and/or FVIII deficiency	Yes (severity expansion)
Inheritance			
Parental mosaic transmission	1 of 21	Molecularly confirmed	Yes
Biochemical			
Abnormal transferrin glycosylation	20 of 21	Core feature	No
Normal transferrin profile	1 of 21	Does not exclude diagnosis	Yes
Fibroblast hypoglycosylation	6 of 6 tested	Universal when tested	No

**Table 3:** Comparison of autosomal-dominant and autosomal-recessive STT3A-related congenital disorders of glycosylation. Summary of genetic, biochemical, and clinical features that distinguish autosomal-dominant from autosomal-recessive STT3A-associated congenital disorders of glycosylation, integrating published reports with data from the present study. Features highlight differences in variant location, pathogenic mechanism, effects on *STT3A* and OST-A function, and patterns of clinical severity and phenotypic variability.

	Autosomal-Dominant STT3A-CDG	Autosomal-Recessive STT3A-CDG
Variant location	Highly conserved residues within the catalytic/active site of STT3A	Variants distributed outside the dominant-negative catalytic hotspot
Genetic/pathogenic mechanism	Dominant-negative disruption of OST-A catalytic activity	Loss of function due to reduced *STT3A* abundance and impaired N-glycosylation
Effect on *STT3A* protein levels	Near-normal *STT3A* mRNA and protein levels	Reduced *STT3A* abundance or function
Impact on OST-A function	Qualitative impairment of catalytic activity despite presence of wild-type protein	Quantitative reduction of overall OST-A activity
Core clinical features	Short stature, craniofacial dysmorphism, skeletal anomalies, muscle cramps, variable neurodevelopment	Severe neurodevelopmental impairment with multisystem involvement
Neurodevelopmental severity	Variable; intellectual disability in ~50%, many with preserved or progressive function	Severe, early-onset neurodevelopmental phenotype
Musculoskeletal involvement	Prominent (skeletal anomalies, muscle cramps, early-onset osteoarthritis)	Present but less emphasized
Craniofacial features	Macrocephaly and/or subtle dysmorphic features	Dysmorphic facial features
Speech development	Variable	Severe speech impairment, including absent or minimal speech in reported cases
Hematologic abnormalities	Variable; bleeding diathesis reported in some individuals	Factor VIII and von Willebrand factor deficiency reported
Feeding/growth	Variable	Failure to thrive and feeding difficulties
Overall severity	Variable, often milder neurological involvement	Generally more severe with consistent neurological impairment
Phenotypic variability	Marked inter-individual variability	Relatively homogeneous

## Data Availability

Data are available from the corresponding authors upon reasonable request.

## Supplementary Materials

The following supporting information can be downloaded at: https://www.mdpi.com/article/10.3390/biom16030418/s1, Supplementary Table S1. Clinical summary of individuals with autosomal-dominant STT3A-CDG. This table summarizes the demographic, genetic, biochemical, and clinical features of 16 individuals from nine unrelated families with autosomal-dominant STT3A-related congenital disorder of glycosylation (STT3A-CDG). All affected individuals harbor heterozygous missense variants clustered at or near the catalytic site of *STT3A*, identified through inherited or de novo events. Clinical features are presented by domain and highlight the variable but characteristic neuromusculoskeletal phenotype, including developmental delay or intellectual disability, short stature, skeletal dysplasia, muscle cramps, and early-onset osteoarthritis. All individuals demonstrated a type I transferrin glycosylation defect, providing a consistent biochemical marker of disease. The dominant STT3A-CDG phenotype is distinguished from the autosomal-recessive form by the relative absence of severe epilepsy, optic atrophy, and profound neurodegeneration, and by the prominence of musculoskeletal manifestations. Supplementary Table S2. Reported heterozygous *STT3A* variants associated with autosomal-dominant STT3A-CDG. This table summarizes published and newly reported heterozygous *STT3A* variants associated with autosomal-dominant congenital disorder of glycosylation (STT3A-CDG). Variants are listed by study, cDNA and protein change, novelty status, inheritance pattern, and relevant clinical or biochemical notes. Most variants cluster at or near the catalytic site of *STT3A*, consistent with a dominant-negative mechanism affecting oligosaccharyltransferase activity. Recurrent variants, particularly those involving Arg405, highlight a mutational hotspot. Where available, accompanying notes indicate transferrin glycosylation abnormalities, inheritance (including de novo and mosaic events), and phenotypic features. Together, these data expand the allelic spectrum of dominant STT3A-CDG and reinforce the central role of active-site disruption in disease pathogenesis. Supplementary Table S3. Phenotypic features of autosomal-dominant STT3A-CDG stratified by inheritance. Clinical and biochemical features of individuals with autosomal-dominant STT3A-associated congenital disorder of glycosylation stratified by inheritance mechanism (de novo versus inherited autosomal dominant, including parental mosaicism). Features are reported as the number of affected individuals within each subgroup. Variable denominators reflect incomplete testing for selected biochemical assays.

## References

[1] Francisco R., Brasil S., Poejo J., Jaeken J., Pascoal C., Videira P.A., dos Reis Ferreira V. (2023). Congenital disorders of glycosylation (CDG): State of the art in 2022. Orphanet J. Rare Dis..

[2] Tinker R.J., Fisher M., Gimeno A.F., Gill K., Ivey C., Peterson J.F., Bastarache L. (2024). Diagnostic delay in monogenic disease: A scoping review. Genet. Med. Off. J. Am. Coll. Med. Genet..

[3] Lipiński P., Tylki-Szymańska A. (2021). Congenital Disorders of Glycosylation: What Clinicians Need to Know?. Front. Pediatr..

[4] Sparks S.E., Krasnewich D.M., Adam M.P., Bick S., Mirzaa G.M., Pagon R.A., Wallace S.E., Amemiya A. (1993). Congenital Disorders of N-Linked Glycosylation and Multiple Pathway Overview. GeneReviews^®^.

[5] Ferreira C.R., Rahman S., Keller M., Zschocke J., ICIMD Advisory Group (2021). An international classification of inherited metabolic disorders (ICIMD). J. Inherit. Metab. Dis..

[6] Shah R., Eklund E.A., Radenkovic S., Sadek M., Shammas I., Verberkmoes S., Ng B.G., Freeze H.H., Edmondson A.C., He M. (2024). ALG13-Congenital Disorder of Glycosylation (ALG13-CDG): Updated clinical and molecular review and clinical management guidelines. Mol. Genet. Metab..

[7] Shrimal S., Cherepanova N.A., Gilmore R. (2015). Cotranslational and posttranslocational N-glycosylation of proteins in the endoplasmic reticulum. Semin. Cell Dev. Biol..

[8] Kelleher D.J., Gilmore R. (2006). An evolving view of the eukaryotic oligosaccharyltransferase. Glycobiology.

[9] Chang I.J., He M., Lam C.T. (2018). Congenital disorders of glycosylation. Ann. Transl. Med..

[10] Shrimal S., Ng B.G., Losfeld M.-E., Gilmore R., Freeze H.H. (2013). Mutations in *STT3A* and STT3B cause two congenital disorders of glycosylation. Hum. Mol. Genet..

[11] Ghosh A., Urquhart J., Daly S., Ferguson A., Scotcher D., Morris A.A.M., Clayton-Smith J. (2017). Phenotypic Heterogeneity in a Congenital Disorder of Glycosylation Caused by Mutations in STT3A. J. Child Neurol..

[12] Wilson M.P., Garanto A., Pinto e Vairo F., Ng B.G., Ranatunga W.K., Ventouratou M., Baerenfaenger M., Huijben K., Thiel C., Ashikov A. (2021). Active site variants in *STT3A* cause a dominant type I congenital disorder of glycosylation with neuromusculoskeletal findings. Am. J. Hum. Genet..

[13] Veitia R.A., Caburet S., Birchler J.A. (2018). Mechanisms of Mendelian dominance. Clin. Genet..

[14] Ezell K.M., Furuta Y., Oglesbee D., Pivnick E.K., Rinker D., Sheehan J.H., Tinker R.J., Hamid R., Cogan J.D., Rives L. (2024). Review and metabolomic profiling of unsolved case reveals newly reported autosomal dominant congenital disorder of glycosylation, type Iw formerly thought to only be an autosomal recessive condition. Mol. Genet. Metab. Rep..

[15] Meng L., Fang Z., Jiang L., Zheng Y., Hong S., Deng Y., Xie L. (2025). Heterozygous pathogenic *STT3A* variation leads to dominant congenital glycosylation disorders and functional validation in zebrafish. Orphanet J. Rare Dis..

[16] Furuta Y., Tinker R.J., Hamid R., Cogan J.D., Ezell K.M., Oglesbee D., DeBerardinis R.J., Phillips J.A., Undiagnosed Diseases Network (2024). A review of multiple diagnostic approaches in the undiagnosed diseases network to identify inherited metabolic diseases. Orphanet J. Rare Dis..

[17] Sivadas A., Moore K., Ezell K., McMinn A., Furuta Y., Bunick C.G., Cassini T., Hamid R., Phillips J.A., Undiagnosed Disease Network (2026). Decoding Genetic Disease Through the Skin: Lessons From the UDN. Int. J. Dermatol..

[18] Tinker R.J., Smith L.M., Bastarache L.A., Ezell K.M., Furuta Y., Hamid R., Cogan J.D., Phillips J.A., Joos K.M., Undiagnosed Diseases Network (2025). The Undiagnosed Diseases Network (UDN) Solves Ocular Syndromic Diagnostic Dilemmas. Am. J. Ophthalmol..

[19] Lam C., Scaglia F., Berry G.T., Larson A., Sarafoglou K., Andersson H.C., Sklirou E., Tan Q.K.G., Starosta R.T., Sadek M. (2024). Frontiers in congenital disorders of glycosylation consortium, a cross-sectional study report at year 5 of 280 individuals in the natural history cohort. Mol. Genet. Metab..

[20] Shuey M.M., Stead W.W., Aka I., Barnado A.L., Bastarache J.A., Brokamp E., Campbell M., Carroll R.J., Goldstein J.A., Lewis A. (2023). Next-generation phenotyping: Introducing phecodeX for enhanced discovery research in medical phenomics. Bioinformatics.

[21] Robinson P.N., Köhler S., Bauer S., Seelow D., Horn D., Mundlos S. (2008). The Human Phenotype Ontology: A Tool for Annotating and Analyzing Human Hereditary Disease. Am. J. Hum. Genet..

[22] Nurk S., Koren S., Rhie A., Rautiainen M., Bzikadze A.V., Mikheenko A., Vollger M.R., Altemose N., Uralsky L., Gershman A. (2022). The complete sequence of a human genome. Science.

[23] den Dunnen J.T., Dalgleish R., Maglott D.R., Hart R.K., Greenblatt M.S., McGowan-Jordan J., Roux A.-F., Smith T., Antonarakis S.E., Taschner P.E.M. (2016). HGVS Recommendations for the Description of Sequence Variants: 2016 Update. Hum. Mutat..

[24] Veitia R.A. (2007). Exploring the Molecular Etiology of Dominant-Negative Mutations. Plant Cell.

[25] Hall P.L., Lam C., Wolfe L., Edmondson A., Acmg Laboratory Quality Assurance Committee (2025). Biochemical testing for congenital disorders of glycosylation: A technical standard of the American College of Medical Genetics and Genomics (ACMG). Genet. Med. Off. J. Am. Coll. Med. Genet..

[26] Hall P.L., Liedke K., Turgeon C., White A., Pino G.B., Peck D., Studinski A., Gavrilov D., Tortorelli S., Oglesbee D. (2024). Sensitivity of transferrin isoform analysis for PMM2-CDG. Mol. Genet. Metab..

[27] Tinker R.J., Bastarache L., Ezell K., Kobren S.N., Esteves C., Rosenfeld J.A., Macnamara E.F., Hamid R., Cogan J.D., Rinker D. (2023). The contribution of mosaicism to genetic diseases and de novo pathogenic variants. Am. J. Med. Genet. A.

[28] Geiger H., Furuta Y., van Wyk S., Phillips J.A., Tinker R.J. (2024). The Clinical Spectrum of Mosaic Genetic Disease. Genes.

